# *Pleurastrosarcina terriformae*, a new species of a rare desert trebouxiophycean alga discovered by an integrative approach

**DOI:** 10.1007/s00792-019-01108-5

**Published:** 2019-06-21

**Authors:** Tatyana Darienko, Woojean Kang, Aleksander K. Orzechowski, Thomas Pröschold

**Affiliations:** 1 Experimental Phycology and Culture Collection of Algae, University of Göttingen, 37073 Göttingen, Germany; 2 Research Department for Limnology, University of Innsbruck, Mondseestr. 9, 5310 Mondsee, Austria

**Keywords:** *Chlorosarcina*, Desert algae, ITS-2 barcode, Molecular phylogeny, *Pleurastrosarcina*, Species concept

## Abstract

Biological soil crusts of extreme habitats (semi-deserts and deserts) are dominated by cyanobacteria and microalgae. The most abundant taxa are green algae belonging to the classes Chlorophyceae and Trebouxiophyceae. Specimens with sarcinoid-like morphology (cells arranged in packages) represent one group of these microalgae. The genus *Pleurastrosarcina* consists of two species, which were originally described as *Chlorosarcina* (*P. brevispinosa* and *P. longispinosa*). Both species are exclusively found from arid soils. However, these species were only reported few times and probably overlooked especially if no akinetes were present. During studying soil samples collected from different regions of the Atacama desert (Chile), we isolated two strains, which were morphologically similar to both *Pleurastrosarcina* species. The phylogenetic analyses confirmed that they belong to this genus. The ITS-2/CBC approach revealed that both new isolates represent a new species, *P. terriformae*. The comparison with other available strains demonstrated that this new species is not restricted to South America and was also found in coastal area in Europe. The six investigated strains showed a high phenotypic plasticity, which is reflected in the descriptions of several varieties.

## Introduction

Microalgae and cyanobacteria, which often form biological soil crusts, play an important role in arid regions such as semi-deserts and deserts around the world (Büdel et al. 2016 and references therein). Many new genera and species of coccoid green algae isolated from desert soil samples were described (i.e., Fučiková et al. 2014; Darienko and Pröschold 2019). Lewis and Flechtner (2002), Lewis and Lewis (2005) and Flechtner et al. (2013) studied the biodiversity of eukaryotic microalgae from different desert regions in North America. Büdel et al. (2009) investigated the biodiversity of southern African soil crusts. In all these studies, the dominant microalgal group were taxa belonging to two classes of green algae, Chlorophyceae and Trebouxiophyceae. As these investigations have shown, green algae with sarcinoid-like morphology often occur in biological soil crusts. They were classified as *Chlorosarcinopsis* (cell packages surrounded by mucilage and parietal chloroplasts with pyrenoids), *Chlorosarcina* (cell packages without mucilage and parietal chloroplasts without pyrenoids), or *Desmochloris* (cell packages without mucilage and parietal chloroplasts with pyrenoids). Detailed morphological descriptions about *Chlorosarcinopsis*, *Chlorosarcina*, and *Desmochloris* are summarized in Groover and Bold (1969), Chantanachat and Bold (1962), and Darienko et al. (2009), respectively. Phylogenetic analyses of several *Chlorosarcinopsis* and *Desmochloris* species have shown that they belong to the Chlorophyceae (Watanabe et al. 2006) and the Ulvophyceae (Darienko and Pröschold 2009, 2017), respectively. About the genus *Chlorosarcina*, very little is known, because only three species are available in public culture collections and only one species has been investigated in detail. *Chlorosarcina stigmatica* originally described by Deason (1959) was later transferred to *Desmotetra* based on ultrastructural investigations of the flagellar apparatus (Deason and Floyd 1987). The other two species of *Chlorosarcina*, *C. brevispinosa* and *C. longispinosa*, were not studied using an integrative approach.

Both species were described by Chantanachat and Bold (1962) from soil of semi-desert in Arizona and Moab desert in Utah (USA), respectively. Both species form package-like structures, often gathered in pseudofilamentous configurations and differ from other *Chlorosarcina* species by production of ornamented akinetes. *Chlorosarcina longispinosa* characterized by production of akinetes with long and thin bristles/spines (definition of both; see “Discussion”) and multipartite chloroplast structure, whereas *Chlorosarcina brevispinosa* typically produced akinetes with shorter bristles/spines and mature vegetative cells, which are possessed by bilobated chloroplast. Both species have parietal chloroplasts, lack pyrenoids and producing zoospores of *Protosiphon*-type (sensu Starr 1955) without a stigma. Since their original description, both species were only reported few times from arid deserts of different continents (Schwarz 1975; Flechtner et al. 2013).

Deason and Floyd (1987) investigated the zoospore ultrastructure of *C. longispinosa* and found that the zoospores exhibit typical counterclockwise orientation of the flagellar apparatus and metacentric nuclear division similar to the strain UTEX 1181 *Friedmannia israeliensis*. The other species of *Chlorosarcina* (*C. stigmatica*) according to its ultrastructure is a member of the Chlorophyceae (Deason and Floyd 1987; Sluiman and Blommers 1990). Therefore, Sluiman and Blommers (1990) established a new genus *Pleurastrosarcina* with its type species *P. brevispinosa*. They also transferred *C. longispinosa* to this genus. According to Sluiman and Blommers (1990), this genus was classified to the order Pleurastrales (Ulvophyceae). This order also contained *F. israeliensis*, which was later transferred to the newly erected class Trebouxiophyceae by Friedl (1995). The genus *Pleurastrosarcina* escaped the attention of scientists for the long time. Lemieux et al. (2014) demonstrated that *Pleurastrosarcina brevispinosa* is a member of core Trebouxiophyceae and forms a separate lineage, which they called “*Pleurastrosarcina*”-clade.

During our study of soil crusts in Las Lomitas at Pan de Azucar National Park and Private Reserve Santa Gracia (Chile), we isolated two strains of *Pleurastrosarcina* and compared them with available strains of public culture collections using an integrative approach.

## Materials and methods

### Study area

The samples were collected from two different locations of the Atacama desert (Chile). (1) Las Lomitas, the part of Pan de Azucar National Park is located in southern part of the Atacama desert. The National Park is situated along the Pacific coast and located in the zone of large-scale coastal fog locally called as “Camanchaca”. The landscape of Las Lomitas characterized by steep mountain range on the coastal site reaching up to 850 m asl and low hills going inland, which can reach between 400 and 700 m asl. The typical air humidity varies from 80–85% at night to 60–70% during the day. Annual precipitation is less than 13 mm. The detailed characteristic about climate, water regime, and landscape of the studied site is provided in Lehnert et al. (2018), (2) Private Reserve Santa Gracia is located in the transition zone of semi-arid/mediterranean to arid regions. The landscape is characterized by inland mountain ranges reaching up to 740 m asl with annual precipitation between 70 and 80 mm.

### Sampling, isolation, cultivation and morphological observation

The two samples of red and yellow crusts were collected in March 2017. The surface of soil crust around 25 cm^2^ and 1–2 cm deep was collected in sterile plastic containers. The samples were transported to the lab and were stored by − 25 °C. For establishing of enrichment cultures, 5–7 pieces of 1 cm^2^ of soil crust were placed in a Petri dish containing Bold Basal Medium (medium 26 in Schlösser 1997) and BG11 (medium 20 in Schlösser 1994). The cultures were incubated at 23 °C, 12:12 h dark:light regime (photon flux rate up 50 μmol m^−2^ s^−1^). The cultures were isolated using Pasteur pipette method (Pringsheim 1946). The light microscopical observations of the cultures were done after 2–3 weeks of growth using Olympus BX60 microscope (Olympus, Tokyo, Japan) equipped with digital camera Prog Res C14 plus (Jenoptik, Jena, Germany). Micrographs were taken using the Prog Res Capture Pro imaging system (version 2.9.0.1, Jenoptik, Jena, Germany).

### DNA extraction and PCR

The two new isolates were compared with the authentic strains of *Pleurastrosarcina longispinosa* (UTEX 1183) and *P. brevispinosa* (UTEX 1176) as well as two strains (SAG 34.83 and ASIB S166), which were isolated by Schwarz (1975) from soil collected Isle of Lavsa, Croatia. The PCR was performed using the MyTaq DNA Polymerase and MyTaq Reaction Buffer (Bioline, Luckenwalde, Germany). The SSU and ITS rDNA were amplified in two overlapping amplicons using green algal-specific primers EAF3 (Marin et al. 2003) and G800R [5′ CATTACTCCGGTCCTACAGACCAACAGG 3′] and G500F [5′ GAATGAGTACAATCTAAACCCCTTAAC 3′] and ITS055R (Marin et al. 2003). PCR products were purified using the MSB^®^ Spin PCRapace (STRATEC Molecular, Berlin, Germany) following the instructions provided by the manufacturer.

### Phylogenetic analyses

The new sequences were assembled using the program SeqAssem (Hepperle 2004). The SSU sequences were included in a data set of the Trebouxiophyceae containing representatives of all lineages known for this class. All sequences were aligned according to their secondary structures. The alignment (1747 bp of 58 taxa) was used for phylogenetic analyses. To determinate the evolutionary model that fits best for the data set, the program Modeltest 3.7 (Posada 2008) was used. Considering the results of these tests, the best model was selected by the Akaike information criterion (Akaike 1974). The GTR model with a proportion of invariable sites (I), and gamma shape parameter (G) was used for calculation of the phylogenetic tree. The significance of the presented tree topology was tested using the bootstrap methods by distance [neighbor-joining (NJ) using the GTR + I+G model], parsimony (MP), and maximum likelihood (ML, using the GTR + I+G model). For all calculations, the program PAUP* (version 4.0b164; Swofford 2002) was used. In addition, Bayesian analysis was conducted using the program MrBayes (version 3.2.3; Ronquist et al. 2012).

Along with the SSU rDNA sequences, the complete ITS regions of the six strains were analyzed to detect compensatory base changes (CBCs). The secondary structures of ITS-1 and ITS-2 were folded using the program Mfold (Zuker 2003).

To check if the newly sequenced strains are widely distributed, we analyzed the V4 and V9 regions of the SSU as well as the ITS-2 using the BLAST N search approach (100% coverage, > 97% identity; Altschul et al. 1990).

## Results

### Molecular phylogeny and species delineation of *Pleurastrosarcina*

**Fig. 1:**
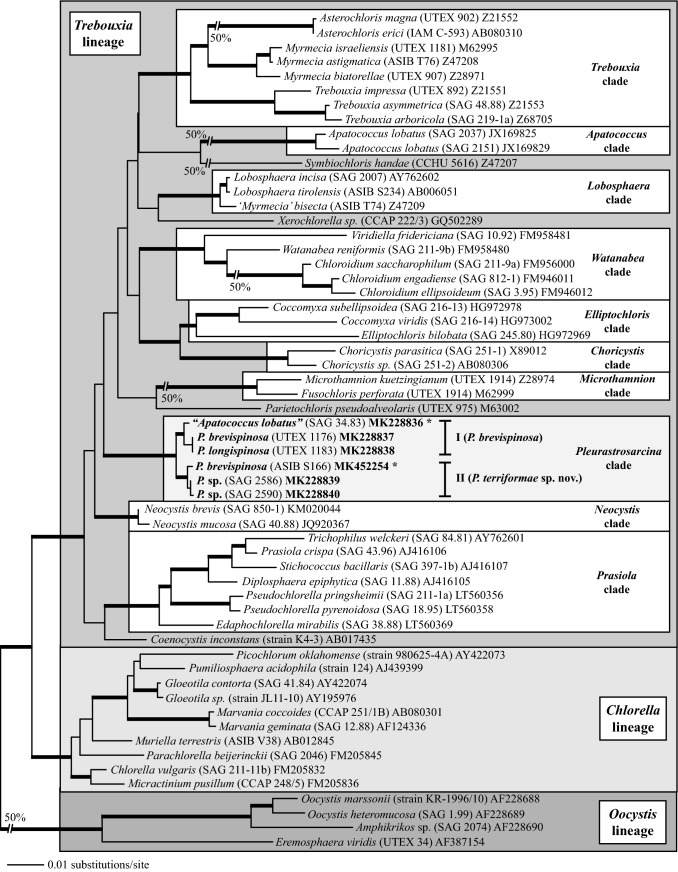
Molecular phylogeny of the Trebouxiophyceae based on SSU sequence comparisons. The phylogenetic tree shown was inferred by maximum likelihood method based on a data set of 1747 aligned positions of 58 taxa using PAUP 4.0b164. For the analysis, the GTR + I+G model (base frequencies: A 0.2461, C 0.2231, G 0.2758, T 0.2550; rate matrix: A–C 0.9868, A–G 2.1737, A–T 0.8540, C–G 1.4063, C–T 6.6452, G–T 1.0000) with the proportion of invariable sites (*I* = 0.6288) and gamma distribution shape parameter (*G* = 0.5597) was chosen, which was calculated as best model by Modeltest 3.7. The Bayesian and bootstrap support was calculated using the same settings. The branches in bold are highly supported (Bayesian values > 0.95; bootstrap values > 70%) in all analyses. The strain designations and accession numbers are given after the species names. The accession numbers in bold represent new sequences of this study, and the newly sequenced strains marked with asterisks contain introns (* = group I intron at position 516)

**Fig. 2:**
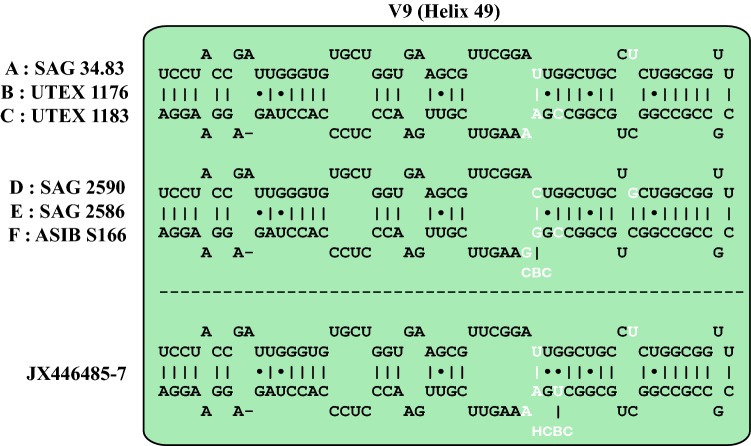
Comparison of the secondary structure of the Helix 49 (V9 region of SSU rRNA) among the species of *Pleurastrosarcina*. The variable regions are highlighted in white letters. The compensatory base change (CBC) and the one-sided CBC (HCBC) are marked

The newly isolated strains (SAG 2586 and SAG 2590) from Chile form together with the authentic strains of *P. brevispinosa* (UTEX 1176) and *P. longispinosa* (UTEX 1183) a monophyletic lineage within the *Trebouxia* lineage of the Trebouxiophyceae. Interestingly, a strain originally assigned as *Apatococcus lobatus* (SAG 34.83) also belongs to this lineage (Fig. 1). The strain ASIB S166 originally identified as *C. brevispinosa* forms together with the two Chilean strains the second subclade within the *Pleurastrosarcina* clade. This lineage is highly supported in all bootstrap and Bayesian analyses. The six strains vary in 15 base positions within their SSU rDNA sequences forming two groups with the *Pleurastrosarcina* clade called groups I and II. The variable positions within the SSU are mostly located in loop regions of different helices. In addition, the strains belonging to group II differ in one compensatory base change (CBC) in Helix 49 (V9 region; Fig. 2) and one hemi-CBC (one-sided CBC) in the helix E23_13 of the SSU secondary structure. The SSU of strain SAG 34.83 only showed one base difference compared to the strains UTEX 1176 and UTEX 1183, but contains a group I intron at position 516. The strain ASIB S166 also has an intron at the same position. The intron sequences of both strains differ from each other. No introns could be discovered in the other strains.

**Fig. 3:**
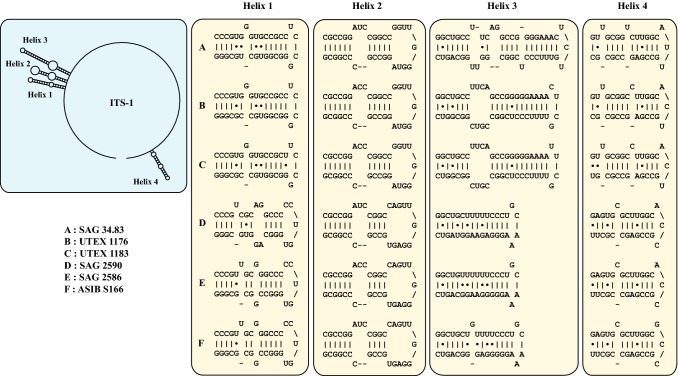
Comparison of the ITS-1 secondary structures among the species of *Pleurastrosarcina*. The line structure of the ITS-1 has been drawn with PseudoViewer3 (Byun and Han 2009)

**Fig. 4:**
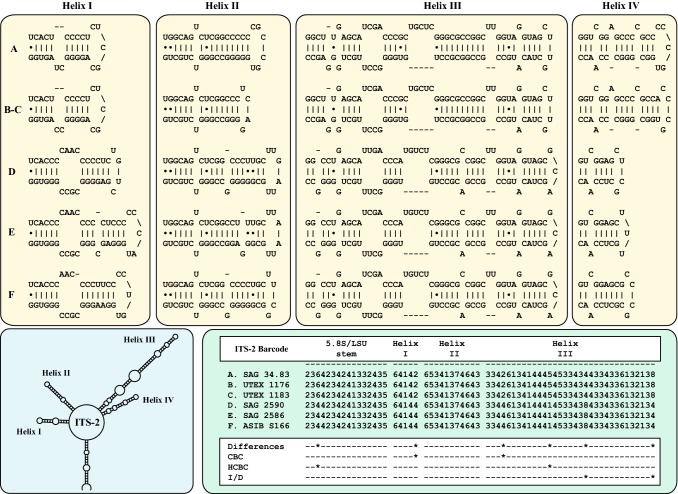
Comparison of the ITS-2 secondary structures among the species of *Pleurastrosarcina*. Extraction of this region and translation into a number code for its usage as barcode. Number code for each base pair: 1 = A–U; 2 = U–A; 3 = G–C; 4 = C–G; 5 = G∙U; 6 = U∙G; 7 = mismatch; 8 = deletion, single or unpaired bases. The line structure of the ITS-2 has been drawn with PseudoViewer3 (Byun and Han 2009). The differences, CBCs, HCBCs, and insertion/deletion (I/D) within the ITS-2 barcodes are marked with an asterisk

To obtain a better resolution among the six investigated strains, we analyzed the secondary structures of ITS-1 and ITS-2. As demonstrated in Figs. 3 and 4, the ITS-1 and ITS-2 showed the typical four helix structures among the six strains. The varying regions are highlighted in white boxes in the figures. The comparison of the strains belonging to the two groups showed that the structures of group I strains are more similar to each other than to those of group II. To decide if the strains belong to one or different species, we used the ITS-2/CBC approach, which were described in detail in Darienko et al. (2016). The ITS-2 barcodes presented in Fig. 4 clearly revealed that the groups I and II represent two different species. Both groups differ in two CBCs and two HCBCs from each other. Additional CBCs and HCBCs can be detected in ITS-1 (Fig. 3) and in the variable region of ITS-2 (Fig. 4).

To find out if these species are widely distributed, we checked the GenBank entries using the BLAST N search algorithm (100% coverage, > 97% identity). We used for this approach the variable regions V4 and V9 of the SSU rDNA, which are commonly used in environmental studies, and the ITS-2. No additional entries using V4 and ITS-2 could be found. Only three sequences (JX446485-7) using V9 were discovered. We compared the V9 (Helix 49) secondary structure of these sequences with our six investigated strains. As shown in Fig. 2, the structures of these sequences only differ in one base, which represents an HCBC.

### Morphological observations

**Fig. 5:**
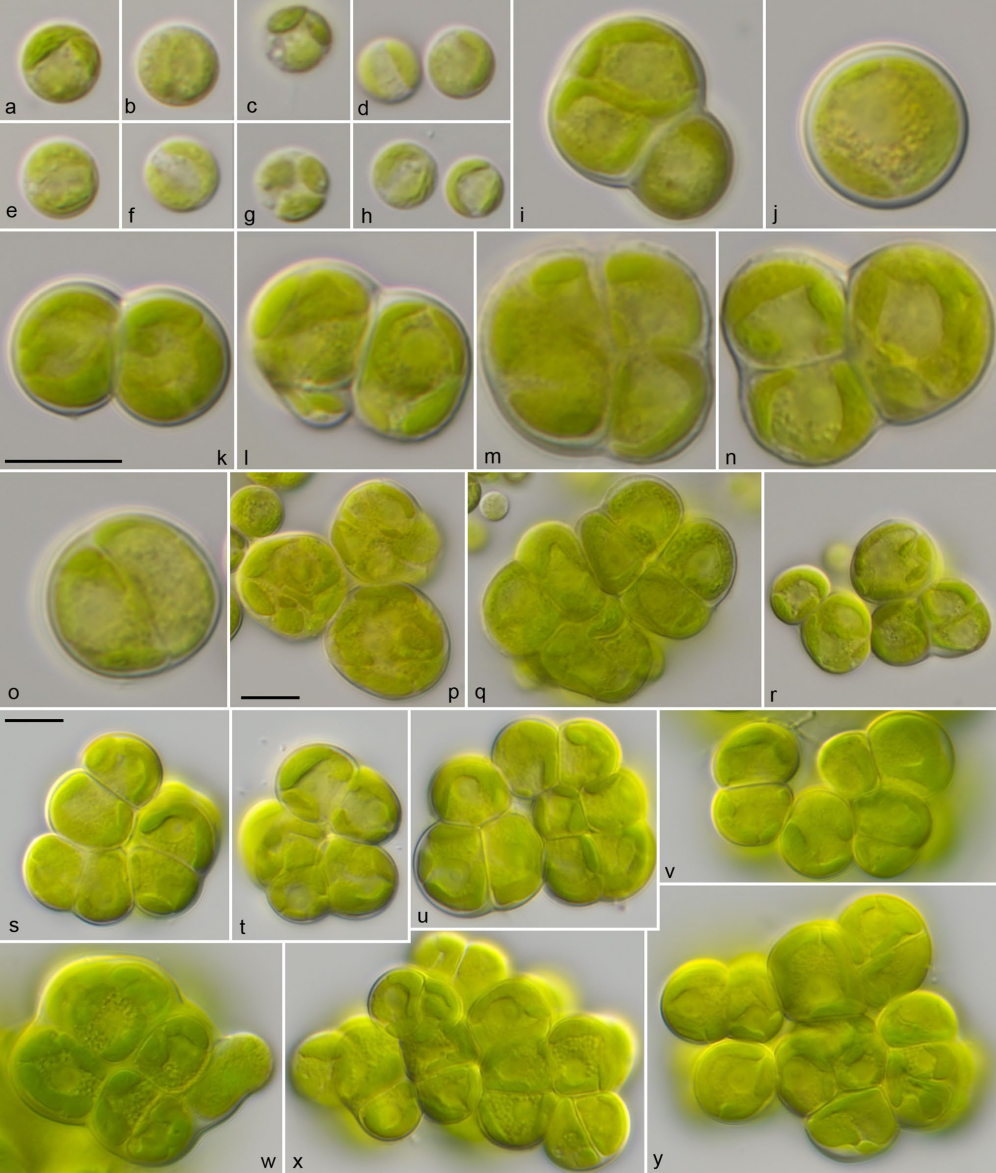
Morphology and phenotypic plasticity of *Pleurastrosarcina*. **a**–**r***P. brevispinosa* var. *schwarzii*, strain SAG 34.83, **s**–**y***P. terriformae* var. *sanctae*-*graciae*, strain SAG 2590. Scale bar: 10 µm

**Fig. 6:**
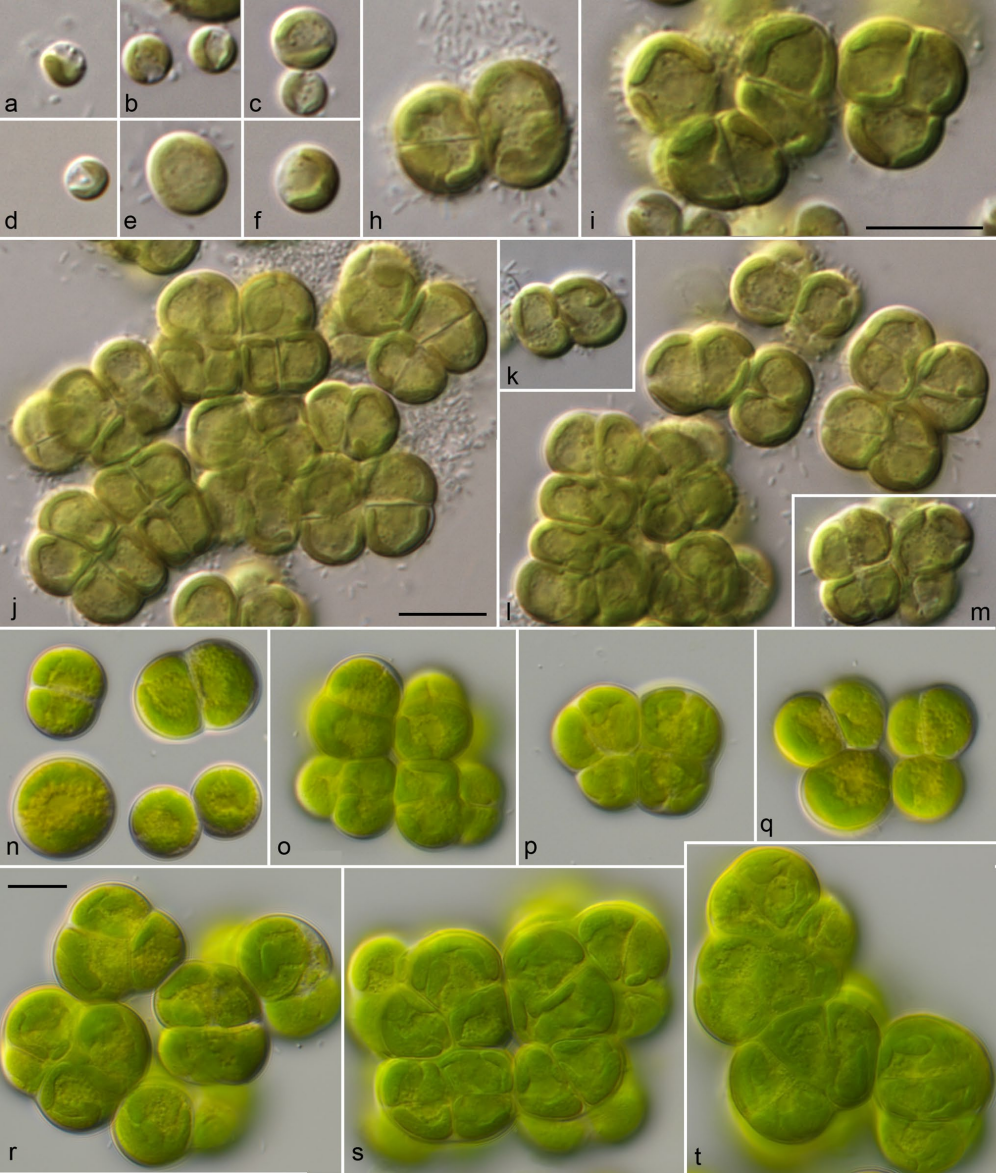
Morphology and phenotypic plasticity of *Pleurastrosarcina*. **a**–**m***P. brevispinosa*, strain UTEX 1176, **n**–**t***P. terriformae*, strain SAG 2586. Scale bar: 10 µm

**Fig. 7:**
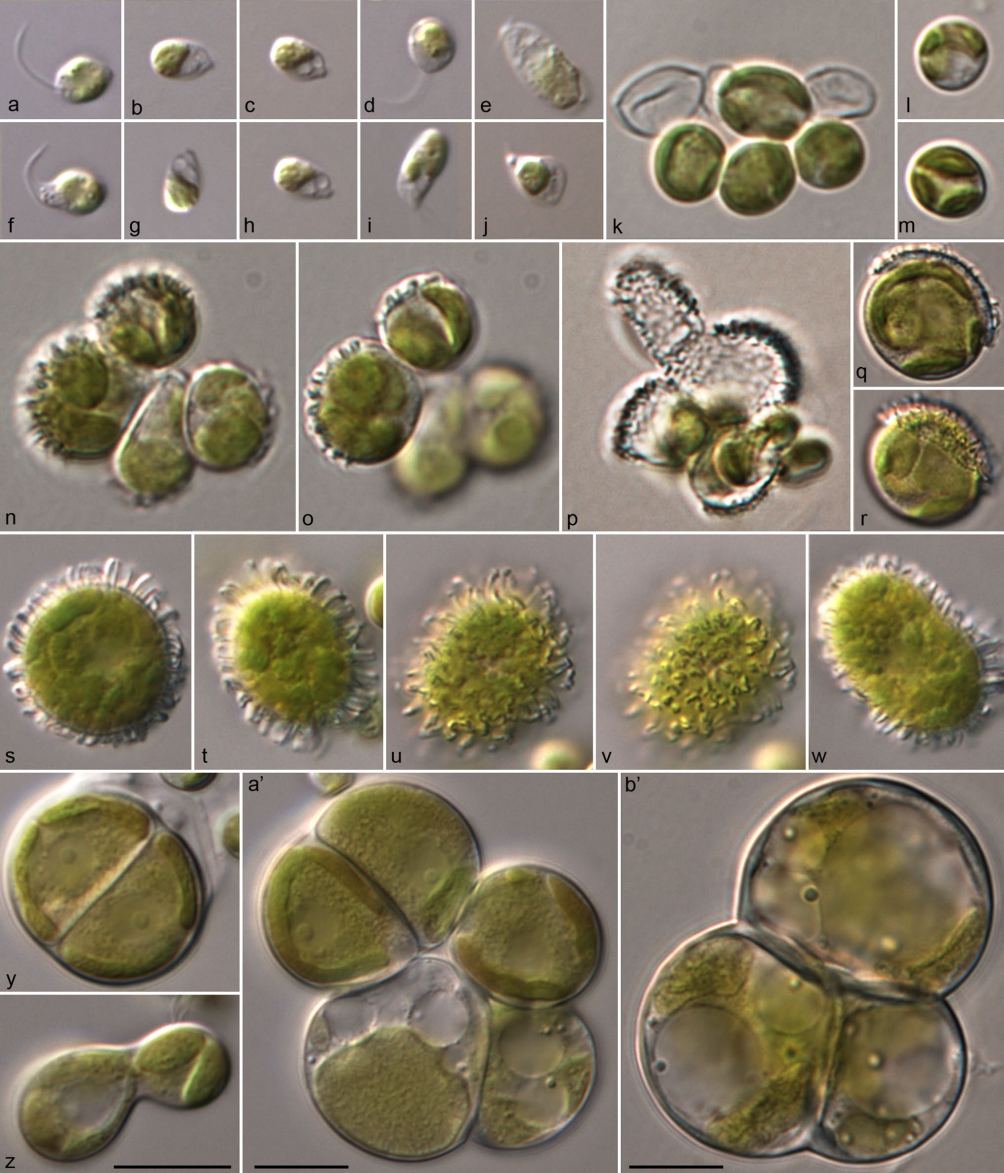
Morphology and phenotypic plasticity of *Pleurastrosarcina*. *P. brevispinosa* var. *longispinosa*, strain UTEX 1183. Scale bar: 10 µm

**Fig. 8:**
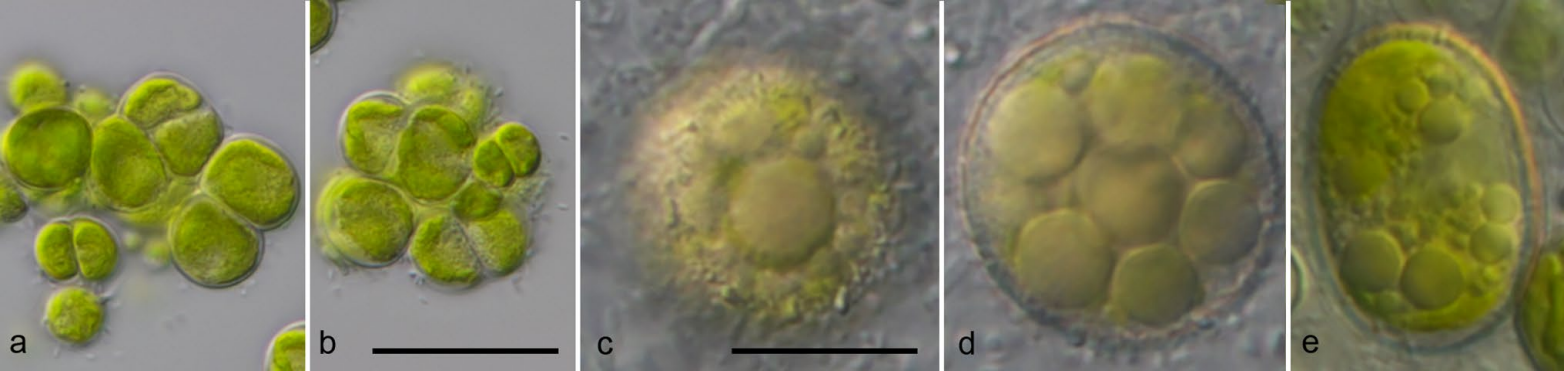
Morphology and phenotypic plasticity of *Pleurastrosarcina*. *P. terriformae* var. *lavsae*, strain ASIB S166. Scale bar: **a**, **b** 30 µm, **c**–**e** 10 µm

For comparison of the morphology, the six strains of *Pleurastrosarcina* were cultivated under identical conditions, as described in “Materials and methods”. Figures 5, 6, 7, and 8 demonstrate that all strains (except of UTEX 1183 and ASIB S166) showed similar morphology (three-dimensional cell packages, bi- to trilobate chloroplast without pyrenoid, and without mucilage surrounding the cell packages). The strain UTEX 1183, authentic strain of *P. longispinosa*, differed by formation of short bristles/spines (2–4 µm long; Fig. 7n–w). The other authentic strain investigated in this study, UTEX 1176 (*P. brevispinosa*), showed no spine formation, which differs to the original description of Chantanachat and Bold (1962). In contrast, the typical spine formation of *P. brevispinosa* (Fig. 8c–e) could be discovered in the strain ASIB S166, which was, therefore, identified as this species by Schwarz (1975). Several experiments (incubation for 2 weeks in darkness in distilled water, transfer to different media including marine media, cultivation at lower temperature of 8 °C) to induce the spine formation failed. Interestingly, both Chilean isolates survive full marine media for more than 3 months without any changes in morphology. Zoospore formation could only be discovered by UTEX 1183 and SAG 34.83. Detailed morphological description of each strain is given below in nomenclature and taxonomical consequences of *Pleurastrosarcina.*

## Discussion

### Molecular phylogeny and phenotypic plasticity of *Pleurastrosarcina*

As demonstrated in Fig. 1, the investigated strains formed a highly supported lineage within the Trebouxiophyceae. Our study confirmed the separation of *C. brevispinosa* and *C. longispinosa* from other species of *Chlorosarcina* based on ultrastructural features (Deason and Floyd 1987), which Sluiman and Blommers (1990) have used for the establishment of *Pleurastrosarcina*. However, Sluiman and Blommers (1990) assigned this genus to the Ulvophyceae, because the Trebouxiophyceae was later described by Friedl (1995). The phylogenetic position of *Pleurastrosarcina* within the Trebouxiophyceae was already demonstrated by Lemieux et al. (2014) based on chloroplast phylogenomic analyses. The comparison of the SSU and ITS rDNA sequences showed that the six investigated strains belong to two species (groups I and II in Fig. 1). The separation into two species is supported by all phylogenetic analyses and the ITS-2/CBC approach (Figs. 1, 2, 3, 4). In contrast, both species were difficult to identify solely by morphology. Chantanachat and Bold (1962) differentiated both species by differences in spine formation and chloroplast structure (bilobated or lobated). Whereas *P. brevispinosa* formed short, at the insertion point broad spines, *P. longispinosa* produced up to 9 µm long, small spines. In contrast as demonstrated in Figs. 5, 6, 7, and 8, only two strains (UTEX 1183 and ASIB S166) showed the typical spine formation. As shown in Fig. 1, both strains belong to different groups indicating that spine formation is a variable feature, which is not suitable for species delineation and not reflected in the phylogenetic analyses. In our morphological comparison, UTEX 1176, the authentic strain of *P. brevispinosa*, showed no spine formation, in contrast to the original description of Chantanachat and Bold (1962). The spine formation could not be induced by different culture conditions as described above. Despite some morphological differences between UTEX 1176 and UTEX 1183 (see Figs. 6a–m, 7), the SSU and ITS rDNA sequences of both strains are identical.

The formation of spines and bristles is not only variable, and it also has different origins and can be differentiated by staining with Calcofluor White (No. 18909; Sigma-Aldrich, Germany) as demonstrated by Schnepf et al. (1980) and Hegewald and Schnepf (1984, 1987). Hegewald and Schnepf (1984, 1987) defined spines, which were developed before cell wall formation, containing fibrils made from cellulose. In contrast, bristles were formed after the cell wall and did not contain cellulose fibrils. Spines can be stained with Calcofluor White, whereas bristles do not show any staining under fluorescence microscope. With Calcofluor White staining, we could demonstrate that all investigated strains contained cellulose fibrils in their cell wall (data not shown); however, if the ornamentations of cell walls (see Figs. 7n–w, 8c–e) represented spines or bristles, this could not decided because of the low abundance of akinetes in the cultures. It seems that the ornamentations were produced after formation of the cell wall. This indicates that *Pleurastrosarcina* produces bristles, but this needs further investigations.

The function of spines/bristles in *Pleurastrosarcina* is unknown. In aquatic habitats, bristle formation can be the response of grazing pressure as demonstrated for *Micractinium* (Luo et al. 2006; Pröschold et al. 2010). Inducible defense against grazing is not studied on terrestrial green algae so far.

Without spine formation or additional morphological features (ultrastructures of zoospores and cell division), *Chlorosarcina*-like algae were difficult to identify at generic level. For example, Gärtner and Ingolić (1989) studied the morphology and ultrastructure of several isolates of *Apatococcus lobatus*, a species usually known as epiphyte of bark of trees and artificial wood constructions such as fences. Only one of their investigated strains was originally isolated from soil (ASIB S183 = SAG 34.83; Schwarz 1975). Despite small differences in morphology, they concluded that the strain SAG 34.83 belongs to *Apatococcus lobatus*. In contrast, our study clearly revealed that this strain is member of *Pleurastrosarcina*, which is a separate lineage to *Apatococcus,* as demonstrated in Fig. 1. This highlights the problematic situation in generic and species delineation based solely on morphology. Green algae-forming cell packages with one chloroplast without pyrenoids are difficult to identify, especially if no further features such as zoospore or spine formation are known. How difficult the identification of taxa with this morphology is, can be demonstrated on the example of *Chlorosarcina stigmatica*. This species was described by Deason (1959) and the ultrastructure of the type strain (UTEX 962) was investigated by Deason and Floyd (1987). Two other strains were isolated by Schwarz (1975) and Trenkwalder (1975), ASIB S163 and ASIB T105, respectively. Sluiman and Blommers (1990) and Gärtner et al. (1988) demonstrated that the ultrastructure of the cell packages of both ASIB strains showed similarities to UTEX 962; however, no pyrenoid could be observed in both strains in contrast to the findings by type strain of *Chlorosarcina stigmatica.* Considering the presence of a naked pyrenoid, Deason and Floyd (1987) transferred UTEX 962 to the newly erected genus *Desmotetra*. Watanabe et al. (2006) demonstrated that UTEX 962 and ASIB T105 belong to Chlorophyceae, but to two different clades and proposed the generic name *Sarcinochlamys* for strain ASIB T105. Several genera with *Chlorosarcina*-like morphology were described: *Chlorosarcina*, *Apatococcus*, *Desmococcus*, *Diplosphaera*, *Coccobotrys*, and others (see Ettl and Gärtner 2014). Most of these genera are typical terrestrial green algae and belong mostly to the Trebouxiophyceae. However, many of these genera need to be taxonomically revised. As consequence, the usage of an integrative approach as in this study is necessary to assign isolates from different habitats to genera and species.

### Ecology and distribution of *Pleurastrosarcina*

All investigated strains were originally isolated from arid habitats. The authentic strains (*P. brevispinosa* and *P. longispinosa*) originated from deserts in Northern America (Chantanachat and Bold 1962), and the two strains isolated by Schwarz (1975) were found in dry soil collected from an island of Croatia. The newly isolated strains from Chilean Atacama desert demonstrated that *Pleurastrosarcina* seems to be widely distributed, but only in dry soils and arid habitats such as deserts. Flechtner et al. (2013) found three isolates from Colorado desert (California, USA). These strains showed the typical *Pleurastrosarcina*-like morphology. The comparison of the V9 region of the SSU rDNA confirmed that these strains belong to *Pleurastrosarcina* (see Fig. 2). Unfortunately, no complete SSU and ITS rDNA sequences are available. Therefore, these isolates cannot be assigned to a species. Summarizing, *Pleurastrosarcina* is a rare genus and restricted to different types of deserts, but distributed at least on three continents (Europe, North, and South America).

### Nomenclature and taxonomical consequences of *Pleurastrosarcina*

As shown in all figures, the investigated strains form two groups, which represent separate species of the genus *Pleurastrosarcina*. This genus originally assigned to the Ulvophyceae belongs to the Trebouxiophyceae and form an own lineage. The group I contains the authentic strains of *P. brevispinosa* and *P. longispinosa* as well as a strain originally assigned as *Apatococcus lobatus*. The two isolates from Chile together with strain ASIB S166 originally identified as *Chlorosarcina brevispinosa* represent the group II. Our new findings require an emendation of the genus *Pleurastrosarcina* and its type species and a description of *P. terriformae* as a new species. In addition, we propose several varieties to reflect the morphological variability within the two species:

***Pleurastrosarcina*** Sluiman & Blommers 1990, Arch. Protistenkd. 138: 189.

**Emended description**: Trebouxiophycean green algae. Cells solitary and spherical compressed in packages. Chloroplasts parietal without pyrenoids. Asexual reproduction by fragmentation of packets or by biflagellated zoospores without cell wall. Zoospores with counterclockwise basal body orientation. Type species: ***P. brevispinosa*** (Chantanachat & Bold) Sluiman & Blommers.

***Pleurastrosarcina brevispinosa*** (Chantanachat & Bold) Sluiman & Blommers 1990, Arch. Protistenkd. 138: 189; holotype: NY03049865 = Chicago 1001962 (**Fig.** **6**a–m).

**Basionym**: *Chlorosarcina brevispinosa* Chantanachat & Bold 1962, Univ. Texas Publ. 6218: 40–41.

**Emended description**: Cells arranged in packages forming pseudofilamentous structures without mucilage. Vegetative cells are oval or spherical, 7.0–9.0 µm in the diameter. Chloroplast bilobated or sometimes trilobated, without pyrenoid. Nucleus is large and good visible. Four-cell packages are 12.0 × 14.0 µm. Reproduction by aplanospore production. Aplanosporangia contain 4–8 spores. Aplanospore are spherical or oval, 4.0–7.0 µm in diameter, containing bilobated chloroplast. Akinetes were not observed. SSU and ITS rDNA sequences (GenBank: MK228837).

Authentic strain: UTEX 1176.

***Pleurastrosarcina brevispinosa*var.*longispinosa*stat. nov.** (**Fig.** **7**)

**Basionym**: *Chlorosarcina longispinosa* Chantanachat & Bold 1962, Univ. Texas Publ. 6218: 42; holotype: NY03049866 = Chicago 1001961.

**Synonym**: *Pleurastrosarcina longispinosa* (Chantanachat & Bold) Sluiman & Blommers 1990, Arch. Protistenkd. 138: 189.

**Emended description**: Cells form sarcinoid-like packages without mucilage. Solitary vegetative cells are 12.0–18.0 µm in diameter. Chloroplast bilobated or cut into several lobes, without the pyrenoid. Nucleus is located in the middle of cell and is good visible. Cytoplasm contains many droplets (probably oil). Cell wall is always thin. Cells form two-to-four-cell packages, which become later three-dimensional structures. In old cultures, cells can reach 25.0 µm in diameter and contain several large vacuoles. The asexual reproduction by zoospore and aplanospore formation. The zoospores and aplanospores are produced in akinetes. Akinetes are spherical or oval, 15.0–18.0 µm in diameter and covered by 3.0–4.0 long spines. Each akinete produce four or eight daughter cells (zoospores or aplanospores). The daughter cells are released by rupture of the mother cell. The remains of akinete cell wall can be observed for the long time in culture. Sometimes, the young cell derived from aplanospores has a rest of the mother cell wall which remains in the top of the cell. The zoospores are of *Protosiphon* type (without cell wall and become round after a short period of moving), 6.0 × 4.0–9.0 × 4.0 µm in size, have parietal bilobated chloroplasts, anterior nuclei, and are biflagellate, possessing two anterior contractive vacuoles. The young cells are spherical, 7.0–8.0 µm in diameter, with bi- or three-lobated chloroplasts. SSU and ITS rDNA sequences (GenBank: MK228838).

Authentic strain: UTEX 1183.

***Pleurastrosarcina brevispinosa*var.*schwarzii*var. nov.** (**Fig.** **5****a–r**)

**Description**: Cells form two- later three-dimensional sarcinoid-like packages. Four-cell packages are 18.0 × 20.0 until 16.0 × 28.0 µm in size. Eight-cell packages can reach 32.0 × 38.0–23.0 × 28.0 µm in size. Cells are spherical if solitary, 11.5–16.0 µm in diameter. Cell wall is relatively thick without ornamentation. Chloroplasts lack pyrenoids and are lobated into two, three, and sometimes more lobes and are pleated. Cell nucleus is large and good visible, often surrounded by small numerous droplets. Asexual reproduction by aplanospores. Aplanosporangia usually contain 4–8 spores, which are released through the rupture of the cell wall. Young cells are broadly ellipsoidal or spherical, 5.0–7.0 µm in diameter, containing usually bilobated chloroplasts. Sometimes, the aplanospores remain in the mother cell and form the packages of the second generation. The liberation of the daughters cells through rupture of the sporangia cell wall. The remains of sporangium cell walls often stick on the surface of the young cells for a long time. Reproduction by zoospore production or sexual reproduction were not observed. SSU and ITS rDNA sequences (GenBank: MK228838).

**Authentic strain**: SAG 34.83.

**Holotype** (designated here): The authentic strain SAG 34.83 cryopreserved in a metabolic inactive state at the Culture Collection of Algae (SAG), University of Göttingen, Germany.

**Etymology**: This variety was named in honor to the isolator of the strain, Dr. Kurt Schwarz.

**Type locality**: Croatia, Dalmatia, Kornati National Park, Isle Lavsa, soil collected near the coast.

Comment: The three strains of *P. brevispinosa* vary in their morphology (different sizes and spine formation) and were, therefore, described as different varieties of this species.

***Pleurastrosarcina terriformae*sp. nov. (Fig.** **6****n–t)**

**Description**: Cells form three-dimensional sarcinoid-like packages. Cells are broadly ellipsoidal or spherical if solitary, 12.0–23.0 µm in diameter. Multicellular packages often arranged into short pseudofilamentous structures. Cell wall is usually thin without ornamentation. Chloroplasts lacking a pyrenoid and are lobated into two, three, and sometimes more lobes, pleated. Cell nucleus is large and good visible, often surrounded by small oil droplets. Reproduction by aplanospores. Aplanosporangia usually contain 4–8 spores, which are released through rupture of the cell wall. Young cells are broadly ellipsoidal or spherical, 5.0–7.0 µm in diameter, containing usually bilobated chloroplast. Sometimes, the aplanospores remain in the mother cell and form packages of the second generation, which lead to the formation of three-dimensional structures. The remains of sporangia cell wall often stick on the surface of young cells for the long time. Reproduction by zoospore production or sexual reproduction were not observed. Four-cell packages are usually 20.0 × 22.0 or 18.0 × 23.0 µm in size. The algae stick together in clusters even in 3–4-month-old culture. SSU and ITS rDNA sequences (GenBank: MK228839).

**Authentic strain**: SAG 2586.

**Holotype** (designated here): The authentic strain SAG 2586 cryopreserved in a metabolic inactive state at the Culture Collection of Algae (SAG), University of Göttingen, Germany under the barcode number Z000696760.

**Etymology**: This species was named according to the project EarthShape.

**Type locality**: Chile, National Park Pan de Azucar, Las Lomitas. The culture was isolated from the red crust on soil.

***Pleurastrosarcina terriformae*var.*sanctae*-*graciae*var. nov. (Fig.** **5****s–y)**

**Description**: Cells form three-dimensional sarcinoid-like packages. Cells are broadly ellipsoidal or spherical if solitary, 18.0–23.0 µm in diameter. Chloroplasts lobated into two, three, and more lobes. Nucleus is large and located into the middle of cell. Cells contain many small droplets of oil, which provide the culture yellowish color. Reproduction by aplanospores, which are release after the rupture of sporangia cell wall. Other types of asexual or sexual reproduction were not observed. SSU and ITS rDNA sequences (GenBank: MK228840).

**Authentic strain**: SAG 2590.

**Holotype** (designated here): The authentic strain SAG 2590 cryopreserved in a metabolic inactive state at the Culture Collection of Algae (SAG), University of Göttingen, Germany under the barcode number Z000694521.

**Etymology**: This variety was named according to the origin of this strain, Santa Gracia.

**Type locality**: Chile, National Park Santa Gracia. The culture was isolated from the yellow crust.

***Pleurastrosarcina terriformae*var.*lavsae*var. nov. (Fig.** **8****)**

**Description**: Cell package formation and cell morphology similar to the type variety, but differs by formation of akinetes with spines. SSU and ITS rDNA sequences (GenBank: MK452254).

**Authentic strain**: ASIB S166.

**Holotype** (designated here): The authentic strain ASIB S166 cryopreserved in a metabolic inactive state at the Culture Collection of Algae (SAG), University of Göttingen, Germany under the barcode number Z000696412.

**Etymology**: This variety was named according to the origin of this strain, Isle Lavsa.

**Type locality**: Croatia, Dalmatia, Kornati National Park, Isle Lavsa, soil collected near the coast.

Comment: The three strains of *P. terriformae* vary in their morphology (different sizes, spine formation by the variety *lavsae*) and were, therefore, described as different varieties of this species.
